# Assessment of the Incidence of Hemi-Diaphragmatic Paralysis Following Infraclavicular and Supraclavicular Approaches for Brachial Plexus Block: A Randomized Controlled Study

**DOI:** 10.4274/TJAR.2025.241648

**Published:** 2025-02-11

**Authors:** Aruna Parameswari, Anisha Pauline Paul, Krithika U

**Affiliations:** 1 Sri Ramachandra Institute of Higher Education and Research Department of Anaesthesiology and Pain Medicine, Tamil Nadu, India

**Keywords:** Hemi-diaphragmatic paralysis, infraclavicular brachial plexus block, phrenic nerve palsy, supraclavicular brachial plexus block

## Abstract

**Objective:**

With the regional anaesthetic technique used for brachial plexus block, the phrenic nerve (C3-C5) can be blocked due to its anatomical proximity to the brachial plexus and the effect of a significant volume of local anaesthetic deposited near the nerve roots. The goal of this study was to compare the incidence of hemi-diaphragmatic paralysis (HDP) following infraclavicular and supraclavicular approaches for brachial plexus block, using a low-volume local anaesthetic.

**Methods:**

A total of 60 patients were enrolled in this study: 30 patients were assigned to the supraclavicular brachial plexus block group, and 30 patients were assigned to the infraclavicular brachial plexus block group. Under aseptic precautions and ultrasound guidance, both groups received 20 mL of 0.5% bupivacaine. The diaphragmatic excursion was measured using ultrasound before the block and 2 hours afterward in the postoperative care unit. A reduction in excursion of more than 75% compared with pre-block values was considered complete paralysis, whereas a reduction of 25-75% was considered partial paralysis.

**Results:**

Infraclavicular brachial plexus block (3.33%) had a lower incidence of HDP compared with supraclavicular brachial plexus block (36.66%). The complications in both groups were not significant, and there was no need to use general anaesthesia.

**Conclusion:**

The incidence of phrenic nerve palsy in the supraclavicular and infraclavicular brachial plexus groups was low, with a lower incidence of HDP in the infraclavicular group.

**Main Points**

• Phrenic nerve palsy (PNP) leading to hemi-diaphragmatic paralysis is a well-known consequence of brachial plexus block, with varied incidence depending on whether the block is performed using an anatomical landmark technique without guidance or guided by a nerve stimulator.

• Ultrasound-guided blocks allow for precise administration of local anaesthetic, potentially reducing the volume or dose of the drug needed in clinical practice.

• The ultrasound-guided infraclavicular approach to the brachial plexus block may be safer against PNP compared with the supraclavicular approach.

## Introduction

Regional anaesthetic techniques for upper limb surgeries offer several advantages over general anaesthesia, including the ability for patients to remain conscious, minimize polypharmacy, improve cardiovascular stability, and provide adequate postoperative pain relief.^1, 2^ Due to its rapid onset and higher success rate, the supraclavicular approach to brachial plexus block has been favored by anaesthesiologists for upper limb surgeries over the infraclavicular approach, which is considered more complex.^3^ However, the primary drawbacks of supraclavicular blocks include an increased risk of complications such as unintentional intravascular injections, pneumothorax, phrenic nerve palsy, and Horner’s syndrome. The advent of ultrasound in anaesthesiology has become a valuable adjunct in brachial plexus blocks, leading to renewed interest in the infraclavicular approach.^4^

Phrenic nerve palsy (PNP) leading to hemi-diaphragmatic paralysis (HDP) is a well-known consequence of brachial plexus block, with varied incidence rates. The rates of phrenic nerve paralysis vary widely depending on the approach and technique (multiple injections vs. single injection; “corner pocket” vs. neural cluster; ultrasound-guided vs. nerve-stimulation-guided),^5, 6^ as well as the volume and concentration of the local anaesthetic used.^7^ The incidence of diaphragmatic paralysis following a supraclavicular block has been reported to range from 0% to 67%,^5, 6, 7, 8, 9, 10^ whereas for the infraclavicular approach, the range is 0-26%.^11, 12, 13^

Transient HDP, although generally well tolerated in healthy individuals, can lead to respiratory complications in patients with pre-existing respiratory compromise or significant abdominal obesity.^14, 15, 16, 17^ Traditionally, the diagnosis of PNP is confirmed through real-time fluoroscopy, pulmonary function testing, or chest radiography. In recent times, M-mode ultrasonography has emerged as an innovative imaging technique that measures diaphragmatic excursion by demonstrating paradoxical movement of the diaphragm indicative of paralysis.^18^ Thus, M-mode ultrasonography is an easy, reliable, non-invasive alternative imaging technique, available at the bedside, for assessing diaphragmatic function.^19, 20^

The advantages of using ultrasound to investigate PNP include its speed, ease of use, and accuracy in evaluation while avoiding radiation exposure. It offers high specificity and sensitivity in analyzing diaphragmatic excursion.^21, 22, 23, 24, 25^

This study primarily aims to determine the incidence of HDP due to PNP in contemporary ultrasound-guided supraclavicular brachial plexus block versus infraclavicular brachial plexus block with equal low volumes of local anaesthetic administration. The occurrence of PNP is assessed by measuring diaphragmatic movements using M-mode ultrasonography.

## Methods

Sri Ramachandra Institute of Higher Education and Research Institutional Ethical Committee clearance was obtained (approval no.: IEC/21/JUN/163/23, date: November 14, 2022), and clinical trial registration (CTRI Trial 2022/11/047720) was completed before the study commenced. This prospective, randomized, single-blinded study was conducted in the operating rooms of "Sri Ramachandra Institute of Higher Education and Research" between October 2021 and October 2022.

Patients scheduled to undergo right upper extremity surgery under brachial plexus block were enrolled in this study after providing written informed consent. The inclusion criteria were American Society of Anesthesiologists (ASA) Physical Status I and II elective surgical patients aged 20-60 years who were undergoing upper extremity surgeries and had a body mass index less than 35 kg m²^-1^.

The exclusion criteria were as follows: patients who refused to participate; those who were pregnant; individuals with acute or chronic pulmonary disease, neuromuscular disease, or allergies to local anaesthetics; and patients who experienced a failed block and required conversion to general anaesthesia.

**Figure 1:**
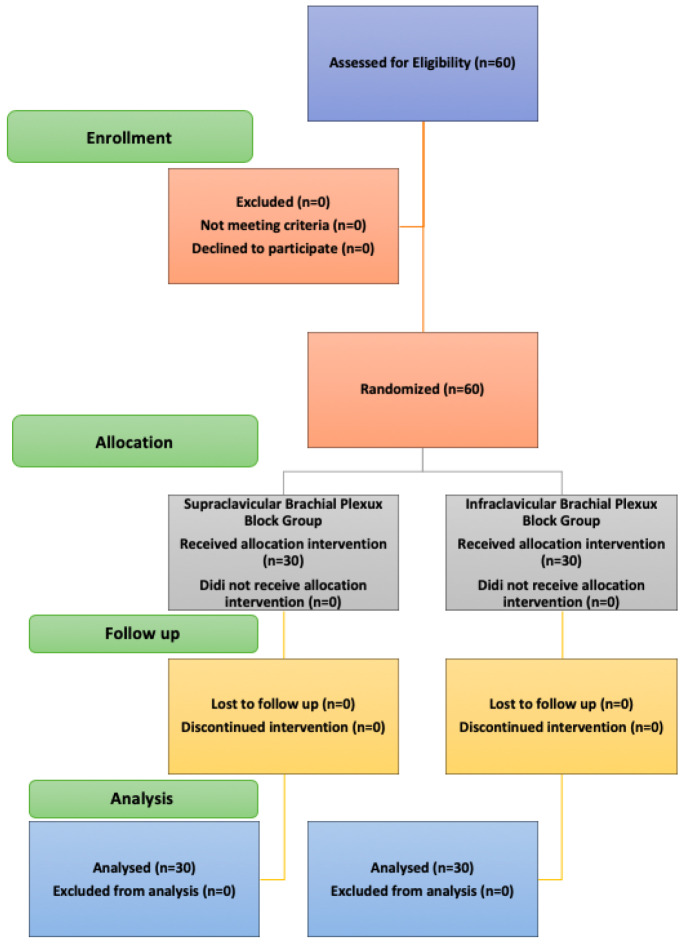
Consolidated Standards of Reporting Trials CONSORT diagram showing the process flow

The principal investigator collected detailed patient histories and performed clinical examinations. The patients were then randomly assigned to one of two groups using computer-generated randomization. Anonymity was maintained by using sealed opaque envelopes numbered sequentially from 1 to 60 (Figure 1). Each envelope was opened and viewed only by the anaesthesiologist performing the block, who was familiar with both supraclavicular and infraclavicular brachial plexus blocks. The patients were unaware of the block group to which they had been assigned. Outcomes were assessed by a different, blinded anaesthesiologist who performed the M-mode ultrasound examination.

All patients were instructed to refrain from oral intake for 8 hours before surgery. Before administering the block, patients were sent to the holding area, where their vital signs were recorded. Baseline measurements of diaphragmatic excursion were taken by the primary investigator, who was blinded to group allocation.

After positioning the patients supine, a low-frequency curved array transducer was used in B-mode to measure diaphragmatic excursion. The liver served as an acoustic landmark to guide the probe medially, cephalad, and dorsally, positioning it between the right mid-clavicular and mid-axillary lines, beneath the right costal border, and focusing on the posterior third of the right hemidiaphragm.

Once optimal images were obtained, the ultrasound machine was switched to M-mode, where the diaphragm appeared as a crisp white, hyperechoic line moving with the respiratory cycle in a slow, smooth up-and-down motion. Under normal resting conditions, during inspiration, the diaphragm moved toward the transducer, resulting in an upward deflection in the M-mode trace. During expiration, the diaphragm moved away from the transducer, producing a downward deflection in the M-mode trace. When the patient was asked to take a deep breath, the distance traveled from the baseline to the point of maximal inspiration was measured in centimeters using digital calipers on the ultrasound machine interface. Three readings were recorded, and the average value was noted.

Afterward, the patient was moved to the operating room, where standard monitors were connected and baseline vital signs were recorded. According to the randomization, either a supraclavicular or infraclavicular brachial plexus block was administered using 20 mL of 0.5% bupivacaine under aseptic conditions and with ultrasound guidance by an independent anaesthesiologist who was not involved in outcome measurement. A supraclavicular block was given after positioning a high frequency linear transducer ultrasound probe at the mid-clavicle, to obtain a short axis view at the junction of the first rib and subclavian artery. The needle was advanced using the “in plane technique” towards the “corner pocket” to inject 10 mL of the local anaesthetic after the appropriate motor response of the hand to neurostimulation, and the additional 10 mL was given in the heart of the plexus. Infraclavicular block was performed after placing the transducer medial to the coracoid process and inferior to the clavicle to visualize the axillary artery. The needle was inserted using the “in plane technique” towards the posterior part of the axillary artery, where 10 mL of local anaesthetic was deposited, and the remaining 10 mL was given lateral to the axillary artery to achieve a U-shaped spread around it after appropriate motor response of the hand to neurostimulation. Following the completion of the surgery, the diaphragmatic excursion was re-evaluated after 2 hours in the postoperative care area by the same primary investigator who conducted the preoperative assessment.

The primary outcome measured was complete HDP, defined as a greater than 75% reduction in mean diaphragmatic excursion compared with pre-block values.^26^ Partial paralysis was defined as a 25-75% reduction in mean diaphragmatic excursion compared with pre-block values.^26^ A mean diaphragmatic excursion of less than 25% was considered an absence of significant diaphragmatic excursion.^26^ The incidence of hemi-diaphragmatic palsy, secondary to ipsilateral PNP, included both complete and partial paralysis cases.

Vital signs, including heart rate, blood pressure, and oxygen saturation, were measured and documented pre- and post-block. Complications such as inadvertent vascular puncture, hematoma, nerve injury, pneumothorax, local anaesthetic toxicity, dyspnea, Horner’s syndrome, and block failure (requiring conversion to general anaesthesia) were also documented. Additionally, the need for light sedation and supplementary local anaesthetic infiltration by the surgeon was recorded.

According to the study by Petrar et al.,^12^ the incidence of complete hemi-diaphragmatic palsy following supraclavicular brachial plexus block was 34%, compared with 3% for infraclavicular brachial plexus block. Based on these findings and assuming a 30% risk difference in the incidence of hemi-diaphragmatic palsy between the groups, a minimum sample size of 26 per group was required to achieve a power of 80% with an α error of 0.05. Four additional patients were included in each group to account for potential dropouts or changes in surgical plans.

Quantitative variables between the two groups were analyzed by comparing the means and standard deviations. Categorical variables were analyzed by frequency and proportion. Mean differences along with their 95% confidence intervals were calculated. The independent samples t-test was used to assess the statistical significance of quantitative variables. Categorical variables were compared between groups using cross-tabulation and percentage comparison. The chi-squared test or Fisher’s exact test was employed to test statistical significance for categorical data. A value of *P* < 0.05 was considered statistically significant. NMaster software version 2.0 was used for statistical analysis.

## Results

A total of 60 patients were recruited and enrolled in this study. They were then randomized and assigned to either the supraclavicular brachial plexus block group or the infraclavicular brachial plexus block group. All 60 patients achieved adequate sensory and motor blockade post-block, without delay in onset and completed the study without conversion to general anaesthesia. None of the patients in either group required sedation or supplementary local anaesthetic infiltration by the surgeon. 20 mL of bupivacaine was sufficient to provide an adequate brachial plexus block with either technique. The demographic data, including age, sex, weight, ASA Physical Classification, and site of surgery, were comparable between the two groups (Table 1).

**Figure 2:**
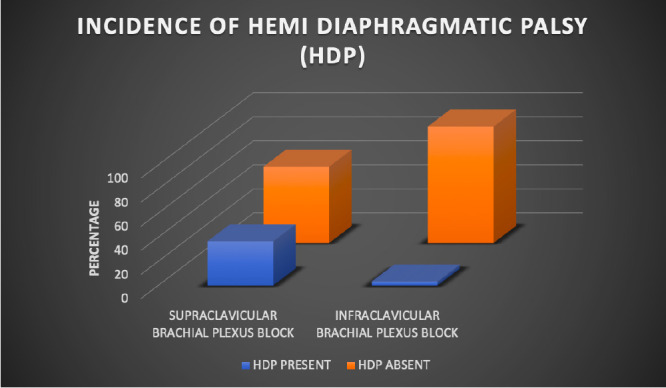
Incidence of hemi-diaphragmatic palsy

The incidence of HDP after brachial plexus block (Figure 2) was significantly lower in the infraclavicular group (3.33%) compared with the supraclavicular group (36.66%) (*P*=0.0047).

A significant difference in the mean diaphragmatic excursion (Table 2) was observed when comparing values before and after the block values between the supraclavicular group (*P*=0.0002) and the infraclavicular group (*P*=0.0000), indicating impaired mean diaphragmatic excursion post-block in both groups. However, when comparing post-block values between the groups, the mean diaphragmatic excursion (Table 2), was more impaired in the supraclavicular group compared with the infraclavicular group (*P*=0.0233).

**Figure 3:**
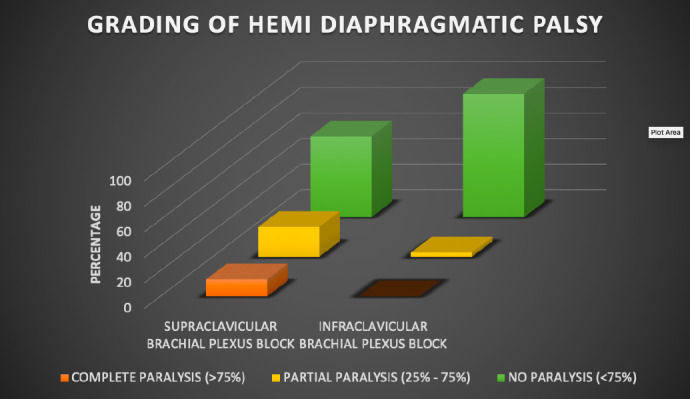
Grading of hemi-diaphragmatic palsy

There was a statistically significant difference in the number of patients with complete paralysis of the diaphragm (4 patients) or partial paralysis (11 patients) in the supraclavicular brachial plexus block compared with the infraclavicular brachial plexus block, which had 1 patient with partial paralysis and 0 patients with complete paralysis (*P*=0.000) (Figure 3, Table 3).

The infraclavicular group had one complication of accidental vascular puncture. However, no other complications related to the brachial plexus block technique, such as nerve injury, pneumothorax, hematoma, breathing difficulty, or Horner’s syndrome, were observed in either group. Additionally, no signs or symptoms suggestive of local anaesthetic toxicity were noted in any of the patients during the study.

## Discussion

Hemi-diaphragmatic paralysis is a common complication following brachial plexus block, particularly with more proximal approaches;^5, 10, 12, 27^ the use of higher volumes of local anaesthetic,^28^ and injections administered within the satellite neural clusters of the brachial plexus.^5, 27^ Although this condition is usually well tolerated in healthy patients, it can be critical in those with poor pulmonary reserve.

Ultrasound-guided regional anaesthesia allows for more precise delivery of the local anaesthetic while reducing the volume and dose required to achieve the desired clinical effect. Therefore, we conducted this prospective, randomized trial to determine the incidence of HDP during ultrasound-guided supraclavicular vs. infraclavicular brachial plexus blocks, using a lower volume of local anaesthetic.

The 20 mL of 0.5% bupivacaine was chosen based on previous studies by Renes et al.^6^ and Duggan et al.,^29^ which demonstrated that using a low volume of local anaesthetic did not result in HDP during ultrasound-guided supraclavicular brachial plexus block while achieving 100% block success.

In our study, the incidence of hemi-diaphragmatic palsy was higher in the supraclavicular group (36.33%) compared with the infraclavicular group (3.33%), with this difference being statistically significant (*P*=0.0047). These results are similar to those of Petrar et al.,^12^ who reported a higher incidence of hemi-diaphragmatic palsy on M-mode ultrasonography with a supraclavicular block (34%) compared with an infraclavicular block (3%). However, a notable difference was that none of our patients with hemi-diaphragmatic palsy experienced breathing difficulties or desaturation, whereas Petrar et al.,^12^ found that patients developed subjective breathing difficulties post-block (43% in the supraclavicular group vs. 75% in the infraclavicular group).

One possible explanation for hemi-diaphragmatic palsy after these brachial plexus blocks is the reverse diffusion of local anaesthetic from the injection site to the level of the cervical nerve roots in the interscalene groove. The local anaesthetic could spread to the phrenic nerve due to anatomical variations or the presence of an accessory phrenic nerve.

Due to the lower volume of local anaesthetic used in our study, none of our patients who developed hemi-diaphragmatic palsy in either group experienced breathing difficulty or desaturation, probably due to the lower concentration of local anaesthetic reverse diffusion from the injection site.

Another study by Johnson and Daniel^26^ concluded that a higher volume of local anaesthetic increased the incidence of hemi-diaphragmatic palsy during ultrasound-guided supraclavicular block. Our findings align with previous research, where the use of a lower volume of local anaesthetic, specifically 20 mL, resulted in a reduced incidence of hemi-diaphragmatic palsy, as assessed by M-mode ultrasonography. However, unlike our study, the researchers employed spirometry-guided FVC and FEV1 measurements to identify the true incidence of ventilatory dysfunction associated with hemi-diaphragmatic palsy. We did not use spirometry measurements because this sequela was not significant in our patients, who were otherwise healthy. Their respiratory rate and oxygen saturation remained stable, likely due to contributions from the opposite diaphragm and accessory muscles.

From the studies mentioned above, it is evident that using ultrasound for brachial plexus blocks, compared with landmark or nerve stimulator-based techniques, does not completely eliminate the risk of phrenic nerve paralysis, whether using the supraclavicular or infraclavicular approach.

A study by Oh et al.^30^ compared ultrasound-guided supraclavicular blocks with costoclavicular blocks (a modification of the traditional infraclavicular approach). PNP was assessed with chest radiography, a less sensitive tool. The incidence of PNP was 2.5% for the costoclavicular block and 3% for the infraclavicular block. However, the costoclavicular block demonstrated better performance in terms of duration and quality of analgesia, and it had less impact on pulmonary function. Therefore, it can be considered an alternative technique for the supraclavicular brachial plexus block.

Diaphragmatic paralysis has traditionally been investigated using chest radiography, fluoroscopic sniff testing, computed tomography, and magnetic resonance imaging.^30^ In this study, we employed a simple, cost-effective, and readily available real-time M-mode ultrasound to measure diaphragmatic excursion; as a paradoxical movement of the diaphragm indicates paralysis. This novel imaging approach can be used to rule out or confirm suspected hemi-diaphragmatic palsy in patients experiencing difficulty breathing or desaturation following a brachial plexus block.

In contrast, the study by Bao et al.^7^ utilized electrophysiological characteristics of the diaphragm to identify HDP. This was based on the amplitude and latency of diaphragmatic compound muscle action potentials, determined by comparing pulmonary function before and after the block. Paralysis was defined as a reduction in the amplitude of the diaphragm CMAP by <50%. Their study also found that a higher volume of local anaesthetic in the supraclavicular block was associated with a higher incidence of diaphragmatic paralysis. We did not use this technique because of the technical challenges associated with electromyography of the hemi-diaphragm. Due to inaccurate placement of subcutaneous needles during diaphragmatic needle electromyography recordings, unreliable measurements of diaphragm CMAP occurred. Additionally, the procedure was uncomfortable for patients. Consequently, in our study, we used only ultrasound, which provided good temporal resolution for detecting and recording rapid movements and was a cost-effective method for observing diaphragm movements at the bedside.

### Study Limitations

Our study has several limitations. First, it did not include patients with pre-existing pulmonary diseases, which could compound the risk of respiratory problems during the perioperative period. Second, further studies are needed to explore the incidence of ventilatory dysfunction associated with hemi-diaphragmatic palsy in obese individuals. Third, the liver’s acoustic window facilitated M-mode ultrasound of the right diaphragm, whereas the splenic acoustic window for the left diaphragm proved less optimal. Thus, the applicability of this approach for left-side brachial plexus blocks requires further investigation. Fourth, patients who underwent continuous peripheral nerve blocks with indwelling catheters were not included in our study. Finally, we did not assess the precise duration of PNP post-brachial plexus block or its recovery in patients with evidence of HDP.

## Conclusion

When a lower volume of local anaesthetic was used, the incidence of HDP due to the involvement of the phrenic nerve was found to be lower in the ultrasound-guided infraclavicular brachial plexus block group (3.33%). Complete paralysis was not observed in any patient who underwent the brachial plexus block via the infraclavicular approach.

## Ethics

**Ethics Committee Approval:** Ethical approval was obtained from the Sri Ramachandra Institute of Higher Education and Research Institutional Ethical Committee (approval no.: IEC/21/JUN/163/23, date: November 14, 2022).

**Informed Consent:** Written informed consent was obtained from the patients.

## Figures and Tables

**Table 1. Demographic Data:** 

**S. No**	**Demographic parameter**	**Supraclavicular brachial plexus block**	**Infraclavicular brachial plexus block**
1.	Sex (Man/Woman)	12/18	15/15
2.	Mean age (years)	42.13±11.15	38.33±9.61
3.	Mean weight (kg)	72.30±12.09	72.87±9.19
4.	American Society of Anesthesiologists Physical Classification (ASA I/ASA II)	11/19	14/16
5.	Site of surgical procedure (Elbow/Forearm/Wrist/Hand)	2/11/15/2	3/13/13/1

The data was represented as frequency or mean (SD).

SD, standard deviation.

**Table 2. Mean Diaphragmatic Excursion:** 

**Mean diaphragmatic excursion (cm)**	**Supraclavicular brachial plexus block**	**Infraclavicular brachial plexus block**	***P* value**
**Pre-block**	4.9 (1.8)	4.8 (1.9)	0.8341
**Post-block**	3.2 (1.5)	3.9 (1.3)	0.0233*
***P* value**	0.0002**	0.000**	-

The data were represented as the mean (SD).

*P values less than 0.05 were considered significant.

**P values less than 0.01 were considered highly significant.

SD, standard deviation.

**Table 3. Incidence and Grading of Hemi-Diaphragmatic Paralysis (HDP):** 

**Incidence of hemi-diaphragmatic paralysis (HDP)**			
**Incidence of HDP**	**Supraclavicular brachial plexus block**	**Infraclavicular brachial plexus block**	***P* value**
HDP present	11 (36.66%)	1 (3.33%)	0.0047**
HDP absent	19 (63.33%)	29 (96.66%)	
**Grading of hemi-diaphragmatic paralysis**			
**Proportion of cases with HDP**	**Supraclavicular brachial plexus block**	**Infraclavicular brachial plexus block**	***P* value**
Complete paralysis (>75%)	4 (13.33%)	0	0.000**
Partial paralysis (25-75%)	7 (23.33%)	1 (3.33%)	
No paralysis (<25%)	19 (63.33%)	29 (96.66%)	

The data were represented as frequency (percentage).

*P* values less than 0.05 were considered significant.

**P values less than 0.01 were considered highly significant.
